# The Perception of Coronary Artery Disease and Cardiac Catheterization in Saudi Arabia: “What the Public Know”

**DOI:** 10.7759/cureus.6570

**Published:** 2020-01-05

**Authors:** Saad Albugami, Faisal Al-Husayni, Lama Bakhsh, Faisal Alhameed, Ahmad Alsulami, Khalid Abumelha, Marwan Balubaid, Maha Al-Harbi, Hani N Mufti

**Affiliations:** 1 Cardiac Sciences / Interventional Cardiology, King Faisal Cardiac Center, King Saud Bin Abdulaziz University for Health Sciences / King Abdullah International Research Center, Jeddah, SAU; 2 Internal Medicine, National Guard Hospital, Jeddah, SAU; 3 Internal Medicine, King Saud Bin Abdulaziz University for Health Sciences, Jeddah, SAU; 4 Nursing, National Guard Hospital, Jeddah, SAU; 5 Cardiac Sciences / Cardiac Surgery, King Faisal Cardiac Center, King Saud Bin Abdulaziz University for Health Sciences / King Abdullah International Research Center, Jeddah, SAU

**Keywords:** coronary artery diseases, cardiac catheterization, awareness, cardiovascular disease, survey, knowledge, perception

## Abstract

Objective

Coronary artery disease (CAD) constitutes a significant health hazard in middle-aged individuals in Saudi Arabia. We sought to assess the level of knowledge of cardiovascular risk factors and describe the perception of coronary intervention among the general population in the city of Jeddah in the western part of Saudi Arabia.

Methods

A cross-sectional study was conducted in the city of Jeddah during the period from April 2019 to September 1, 2019, by using a structured online questionnaire to assess the participants’ awareness of risk factors for CAD and coronary intervention. The survey included questions about socio-demographic data, risk factors of cardiovascular diseases, symptoms of heart attack, knowledge of coronary catheterizations, as well as resources of knowledge about coronary heart disease.

Results

The study included 984 participants. The majority of the participants had university diplomas (78.1%). Only 38.5 % were healthcare workers. Dyslipidemia and smoking were identified by 70.5% and 66.7%, respectively, as a recognized risk factor for CAD. Diabetes was mentioned by 32.1%. Participants without CAD risk factors had a significantly lower level of knowledge regarding the strong association between diabetes mellitus (DM) II and CAD (p-value=0.02). Healthcare professionals had a significantly lower level of knowledge regarding cardiac catheterization as compared to non-healthcare professionals. A higher percentage of healthcare professionals would agree to have cardiac catheterization if indicated (p-value=0.003). Awareness campaigns were the most common source of information for the public.

Conclusion

The current level of knowledge of CAD in the western part of Saudi Arabia is fair. National awareness campaigns are required to improve the level of healthcare education.

## Introduction

Cardiovascular diseases (CVDs) are considered the leading cause of morbidity and mortality globally. They are regarded as the primary cause of extended hospital stays and increased healthcare costs [1-5]. More people die annually from CVDs than from any other cause. An estimated 17.9 million people died from CVDs in 2016, representing 31% of all global deaths. Of these deaths, 85% were due to heart attacks and stroke. Over three-quarters of CVD deaths take place in low and middle-income countries. Based on epidemiological data, cardiovascular diseases will remain the leading cause of mortality and morbidity throughout the next decade [2-7]. The mean age for developing CAD in the Middle East is 10 years younger than the mean age of their western counterparts [2-8]. The prevalence of CAD risk factors among Saudi patients with established CAD is alarming; however, many of these are modifiable risk factors [9-11]. In Saudi Arabia, despite the widespread availability of hospitals with a catheterization laboratory, there are fewer patients treated with a primary percutaneous intervention [12]. The baseline knowledge about CVD among the general population has a significant public impact in developing targeted educational programs [13]. Knowledge of CVDs, its symptoms, and its risk factors have been studied worldwide in various populations [14]. Nevertheless, there is a scarcity of data in Saudi Arabia on the perception of the Saudi public of coronary artery disease (CAD) and coronary catheterization. Therefore, this study aims to examine the level of knowledge and perception of the public towards coronary artery disease and its treatment in an attempt to generate educational programs to reduce the CVDs burden.

## Materials and methods

This was a cross-sectional study conducted at King Saud bin Abdulaziz University for Health Sciences in Jeddah, Saudi Arabia, during the period from April 2019 to September 1, 2019. This was achieved through voluntary participation in an online survey questionnaire that was constructed in the Arabic language. Participants were asked to distribute the survey to their friends and relatives.

The sample size was calculated assuming that we are targeting a sample from only the city of Jeddah, which has an estimated total population of four million inhabitants, with a 99% confidence level and a 5% margin of error; the sample size was determined to be 665 respondents. All volunteer participants who were able to complete the online survey were included. Participants younger than 18 years of age and who could not or did not complete the survey were excluded. The questionnaire was adapted and modified from a previous survey by Almalki et al. [15]. We collected sociodemographic characteristics, assessments of awareness of CAD risk factors, coronary angiography, and CAD treatment. Socioeconomic variables were participants’ age, sex, nationality, marital status, employment, education, and income. Family and personal medical histories, such as diabetes mellitus (DM), hypertension, and dyslipidemia (high cholesterol or triglycerides levels) were collected as well. Awareness of CAD risk factors was assessed through yes or no questions asking whether or not the participants believed certain factors were considered risk factors for CAD. The survey also included questions on symptoms of a heart attack, information about cardiac catheterization, and resources of information about coronary artery disease (See Table 1 for complete survey questions). The study was conducted following the Declaration of Helsinki. It received ethical approval from the Institutional Review Board of King Abdullah International Medical Research Center.

**Table 1:** Survey questions Translated from the Arabic language

Socio-demographics Section	
Age	
	18 to 25
	26 to 35
	36 to 45
	46 to 55
	56 to 65
	Above 65
Gender	
	Male
	Female
Marital Status	
	Married
	Unmarried
Level of education	
	Diploma or university
	High school or less
Health care worker	
	Yes
	No
Smoker	
	Yes
	No
Medical and surgical history	
Diabetes mellitus	
	Yes
	No
Hypertension	
	Yes
	No
Dyslipidemia	
	Yes
	No
Chronic kidney disease	
	Yes
	No
Cardiac disease	
	Yes
	No
History of cardiac cath	
	Yes
	No
History of open-heart surgery	
	Yes
	No
Knowledge about heart attack	
Have you heard of the term “heart attack” before?	
	Yes
	No
Do you know the difference between a heart attack and a cardiac arrest?	
	Yes
	No
Can a person live normally after a heart attack?	
	Yes
	No
Do females have more heart attacks than males?	
	Yes
	No
If someone has a heart attack, does he/she need to go to the hospital by himself/herself and not wait for an ambulance?	
	Yes
	No
Can a heart attack happen to someone younger than 30 years old?	
	Yes
	No
Which of the following is a risk factor of coronary artery disease? (You may choose more than one)	
	Diabetes
	Hypertension
	Dyslipidemia
	Smoking
	Overweight
	Aging
	Sedentary Lifestyle
	Unhealthy diet
	Genetics
	Anger
	Do not know
Which of the following is a symptom of a heart attack? (You may choose more than one)	
	Chest pain
	Shortness of breath
	Jaw or arm pain
	Nausea and vomiting
	Pins and needles sensation in the chest
	Palpitations
	Sweating
	Dizziness
	Do not know
Which of the following is a coronary artery disease diagnostic method? (You may choose more than one)	
	History and physical exam
	Blood tests
	Imaging
	Stress test
	Cardiac catheterization
	Heart biopsy
	ECG
	Do not know
Which of the following is a treatment of coronary artery disease? (You may choose more than one)	
	Anticoagulants
	Thrombolytics
	Vasodilators
	Medications to decrease blood pressure
	Medications to decrease heart rate
	Medications to decrease lipids
	Cardiac catheterization
	Open-heart surgery
	Do not know
Knowledge of cardiac catheterization	
Do you know the difference between diagnostic and therapeutic catheterization?	
	Yes
	No
Who does the cardiac catheterization?	
	Any doctor
	Cardiac surgeon
	Interventional cardiologist
	Do not know
Is contrast used in cardiac catheterization?	
	Yes
	No
	Do not know
What is the access used in cardiac catheterization?	
	Arm or thigh vessels
	Mouth
	Directly through the skin to the heart
	Do not know
Can a cardiac catheterization be a one-day procedure?	
	Yes
	No
	Do not know
Can a stent be inserted using catheterization?	
	Yes
	No
	Do not know
Source of information	
Which of the following is your source of information about coronary artery disease? (You may choose more than one)	
	Doctors
	Internet
	Media and social media
	Campaigns
	Books
	Patients with cardiac disease

Statistical analysis

The results of this survey are mainly descriptive of the perception and knowledge of CAD and cardiac catheterization in a contemporary sample from Jeddah, Saudi Arabia. In this survey, data were reported mostly in the form of frequencies and the percentage of respondents. Categorical variables were analyzed by χ2 (chi-square) or Fisher's exact test as appropriate. The Kruskal-Wallis test was used for ordinal variables. Statistical analysis was performed using the Fisher exact test to evaluate the significance of some of the survey results. Values of p < 0.05 were considered significant. All statistical analysis and assessment of the model’s performance were conducted using the R-Software, version 3.3.0 (R Project for Statistical Computing, Vienna, Austria) [16].

## Results

There were 984 responders to the study survey. The participants were well-educated and relatively young. There were 54.5% females. The majority had university diplomas or higher (78.1%). Two-thirds of the participants were younger than 45 years of age. The prevalence of CAD risk factors among the study group was low; 9.6% had type two diabetes, 9.2 % had hypertension, 7.7% had dyslipidemia, and 18.2 % were smokers. Only 3.9% of the study group had CAD; 38.5% of the cohorts were health care workers (Table 2).

**Table 2:** Respondents demographics (total number of respondents=984)

Variable	Frequency (%)
Age (Years)	
18 to 25	253 (25.7)
26 to 35	198 (20.1)
36 to 45	227 (23.1)
46 to 55	190 (19.3)
56 to 65	103 (10.5)
Above 65	13 (1.3)
Gender	
Male	447 (45.4)
Female	537 (54.6)
Marital Status	
Married	678 (68.9)
Unmarried	306 (31.1)
Educational Level	
Diploma or University	769 (78.2)
Health Care Worker	
Yes	379 (38.5)
No	605 (61.5)
History of Smoking	
Yes	180 (18.3)
No	804 (81.7)
History of Diabetes Mellitus	
Yes	94 (9.6)
No	890 (90.4)
History of Hypertension	
Yes	90 (9.2)
No	894 (90.8)
History of Chronic Kidney Disease	
Yes	3 (0.3)
No	981 (99.7)
History of Dyslipidemia	
Yes	76 (7.7)
No	908 (92.3)
History of Cardiac Disease	
Yes	38 (3.9)
No	946 (96.1)
History of Cardiac Cath	
Yes	48 (4.9)
No	936 (95.1)
History of Open-Heart Surgery	
Yes	5 (0.5)
No	979 (99.5)

The following responses to the questions on heart attack were obtained; 92.2% had heard of the term. Most participants (almost 82%) were able to differentiate between heart attack and cardiac arrest. Three-quarters mentioned that patients who suffered a heart attack could live normally. Almost two-thirds of the participants would not wait for emergency services arrival and would immediately drive themselves to the nearest hospital if they suffered a heart attack. Nearly three-quarters of the participants (72%) believed that a heart attack could occur in a person younger than the age of 30 (Table 3).

**Table 3:** General questions about heart attack

	Response	Frequency	Percent
Heard of heart attack before	No	76	7.7
	Yes	908	92.2
Knows the difference between heart attack and cardiac arrest	No	181	18.3
	Yes	803	81.6
Can a person live normally after a heart attack	No	258	26.2
	Yes	726	73.7
Females have more heart attacks than males	No	293	29.7
	Yes	176	17.8
	Don’t know	515	52.3
Should go to the hospital and not wait for an ambulance when experiencing a heart attack	No	169	17.1
	Yes	598	60.7
	Don’t know	214	21.7
Heart attack could happen in people younger than 30 years old	No	57	5.7
	Yes	710	72.1
	Don’t know	217	22.0

**Figure 1:**
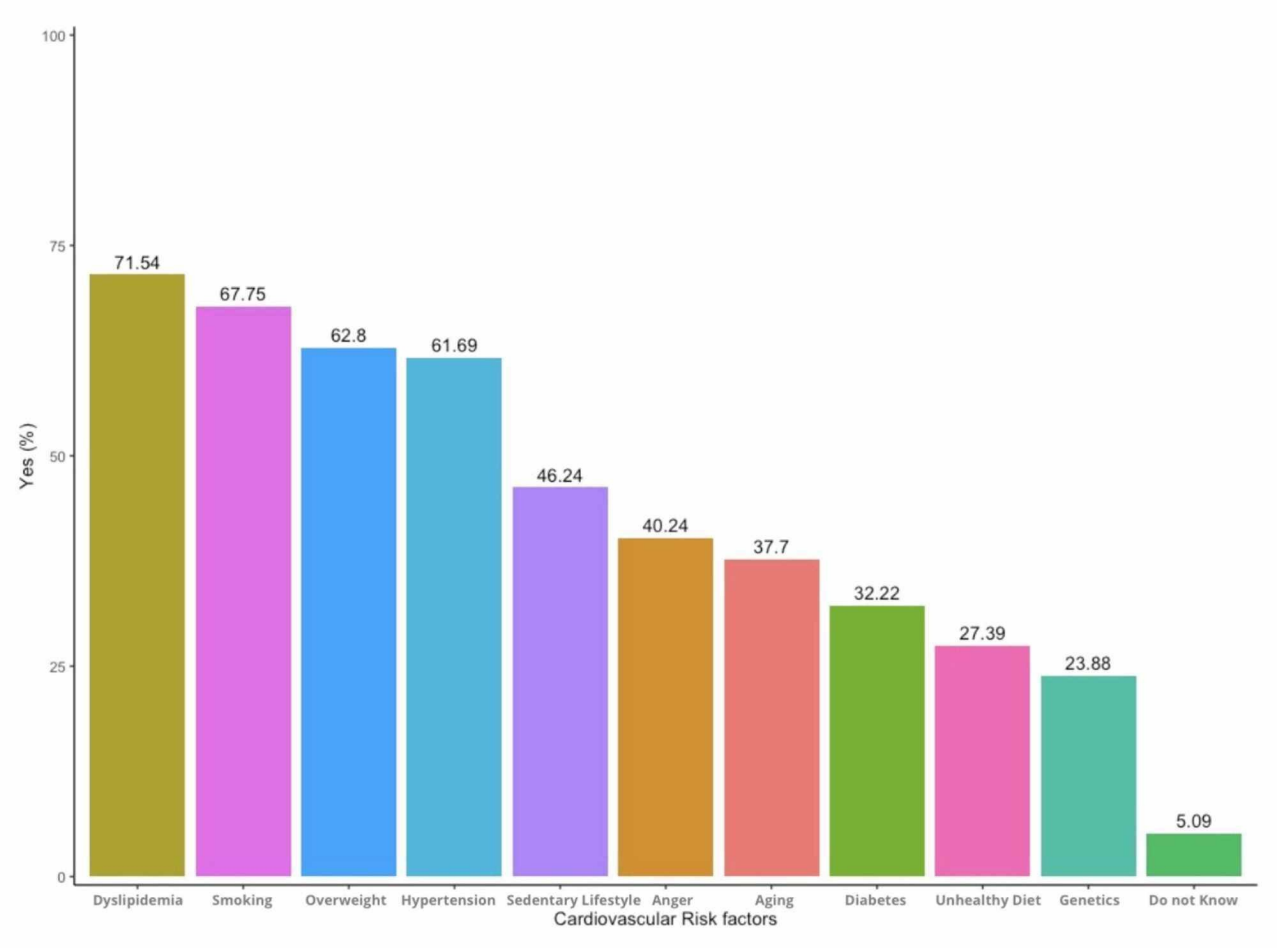
Responses to risk factors of coronary artery disease

Responses to the coronary artery disease risk factors were as follows: 70.5% of the participants agreed that dyslipidemia is a risk factor for coronary artery disease, followed by smoking (66.7%), while diabetes was recognized by only 32.22% of the respondents. Five percent of the participants did not know CAD risk factors, as shown in Figure 1.

**Figure 2:**
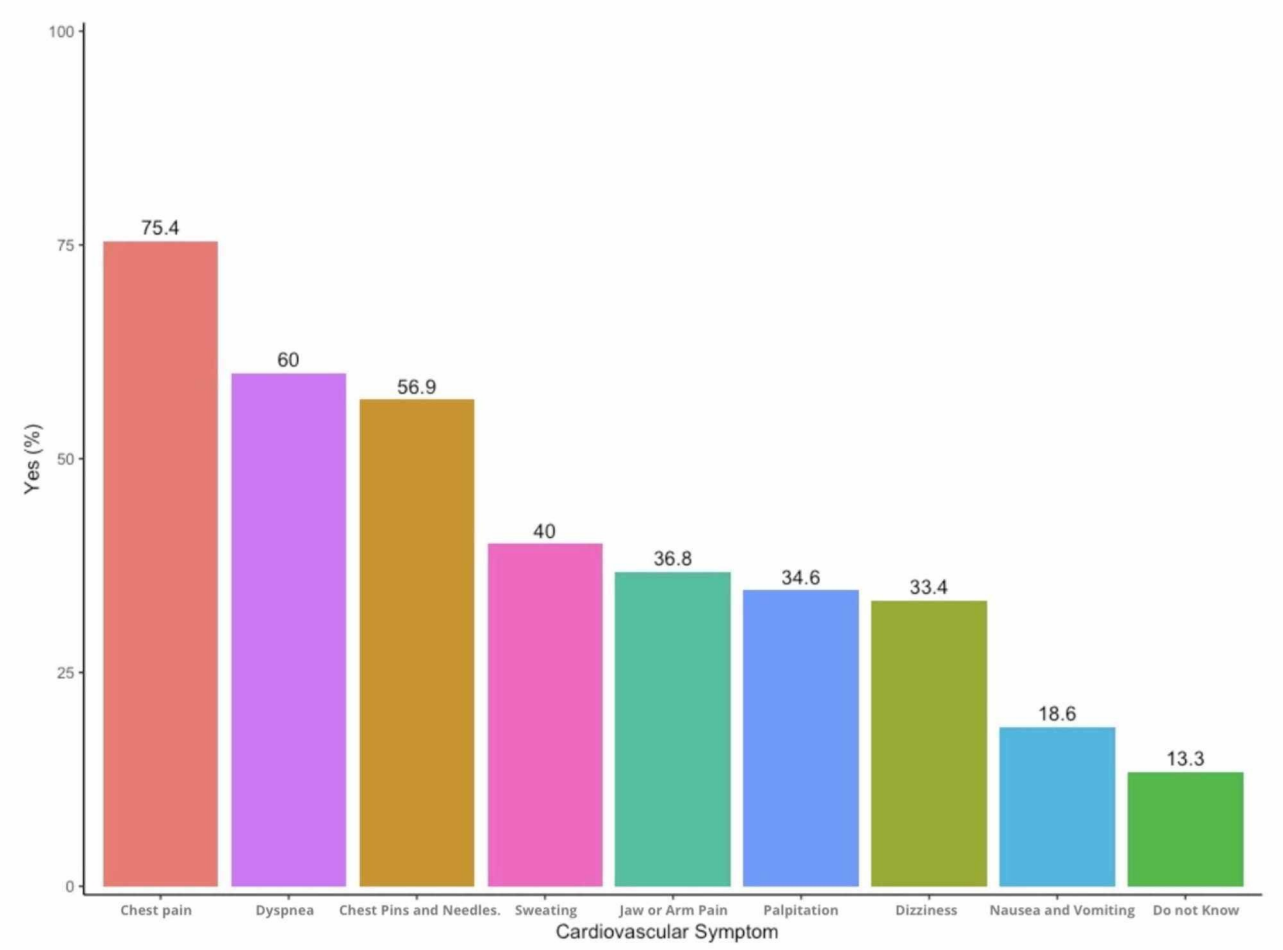
Responses to symptoms of a heart attack

The participants’ perception of the symptoms of a heart attack is shown in Figure 2. Of the responders, 74.3% revealed that chest pain or heaviness is a common symptom of a heart attack, followed by dyspnea (59.1%), loss of consciousness (59.1%), sweating (39.4%), palpitations (34%), and jaw pain (36.2%). Other associated symptoms like nausea and vomiting were mentioned. Only 13% of responders did not know the common symptoms of a heart attack.

**Figure 3:**
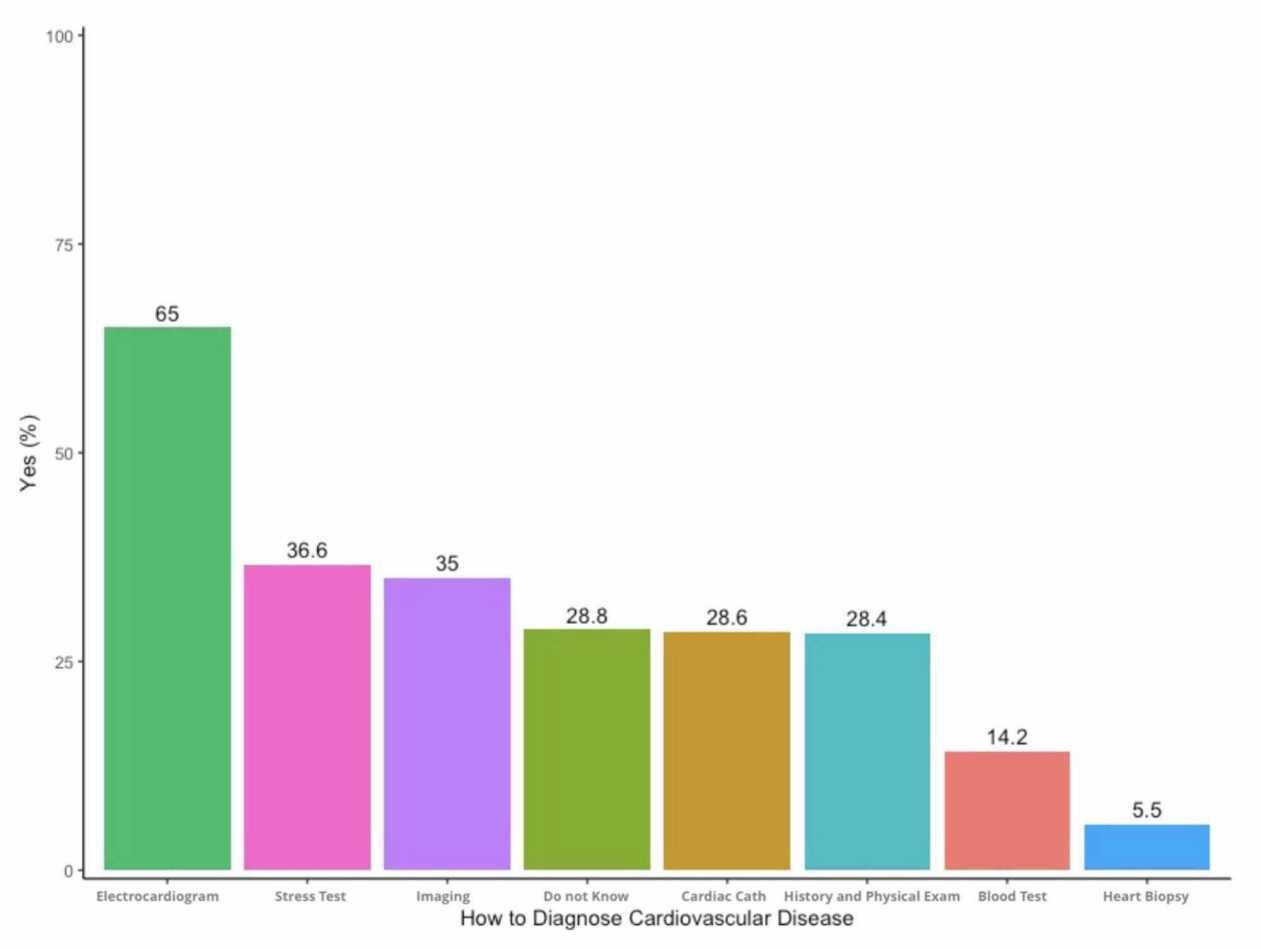
Responses to the diagnosis of coronary artery disease

**Figure 4:**
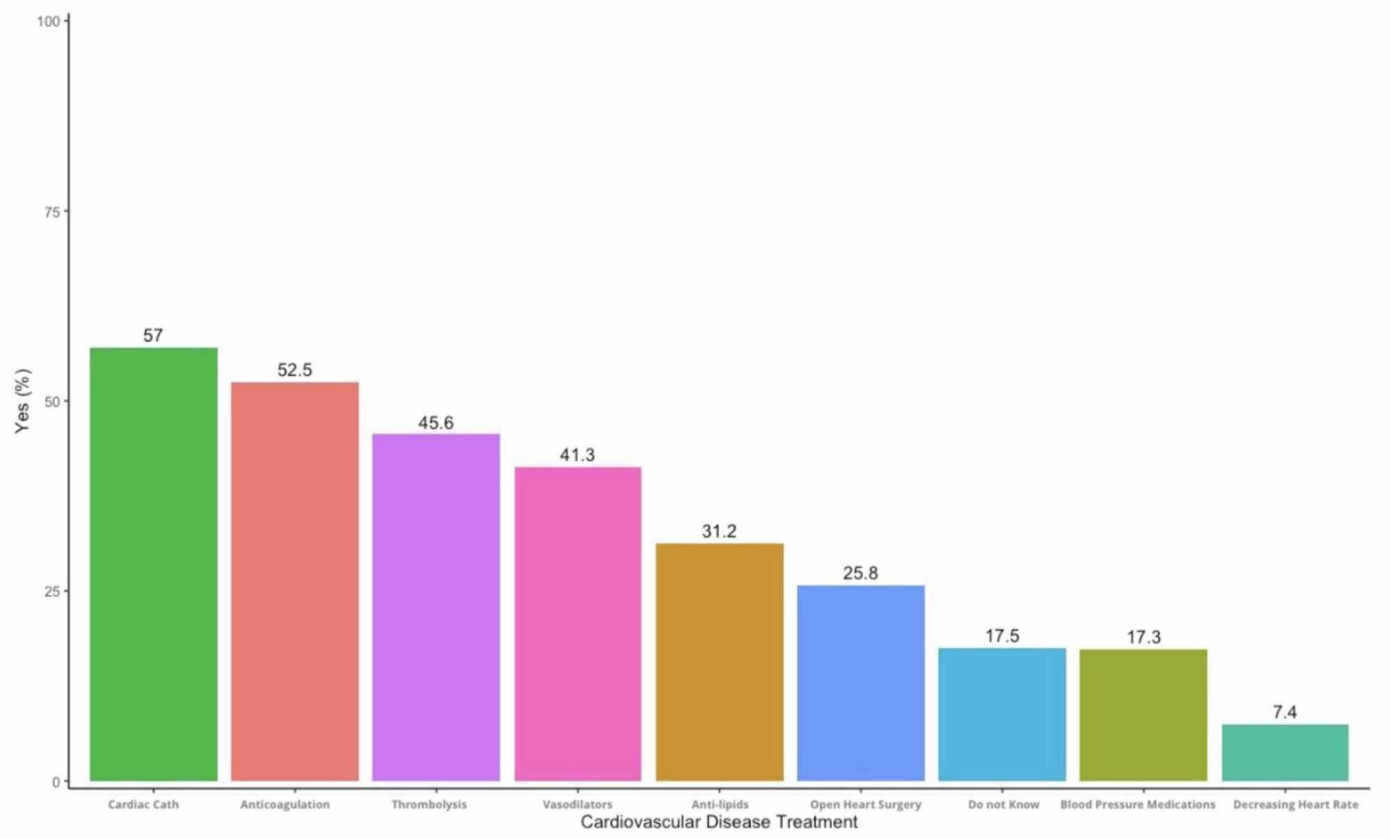
Responses to the treatment of coronary artery disease

Figure 3 shows the perception of the diagnostic tests by the participants. Sixty-four percent mentioned that an electrocardiogram is the best test, followed by a stress test, imaging, and coronary angiography. Fifty-six percent of the cohort agreed that cardiac catheterization and coronary intervention are the best treatment for CAD, followed by anticoagulants (51.8%) and then thrombolytics (44.9) while only 17.2% were unaware of any treatment options (Figure 4).

Thirty percent were able to know the difference between diagnostic and therapeutic catheterization. Out of the 984 participants, 47.3% thought that cardiac catheterization is performed by a cardiac surgeon and 16.5% thought that it is done by an interventional cardiologist. Contrast use during the procedure was mentioned by 61.7%. Catheterization access was reported to be the arm or thigh blood vessels by 60.8%. Five percent thought it is done through the chest wall directly into the heart and 3.4% thought it was through the mouth. The majority of the participants (78.3%) would agree to have a cardiac catheterization if needed while 4.5% did not agree and 17.0% were ambivalent about whether to accept or not (Table 4).

**Table 4:** Responses to questions on cardiac catheterization

	Response	Frequency, (%)
Knows the difference between diagnostic and therapeutic catheterization	No	684 (69.5%)
	Yes	300 (30.4%)
Who does the catheterization	Any doctor	58 (5.8%)
	cardiac surgeon	466 (47.3%)
	Interventional cardiologist	163 (16.5%)
	Don't know	297 (30.1%)
Use of contrast in the catheterization	No	102 (10.3%)
	Yes	274 (27.8%)
	Don’t know	608 (61.7%)
What is the access of the catheterization	Arm or thigh vessels	599 (60.8%)
	Mouth	34 (3.4%)
	Directly through to the heart	50 (5.0%)
	Don't know	301 (30.5%)
Can a catheterization be a one-day procedure	No	310 (31.5%)
	Yes	385 (39.1%)
	Don’t know	289 (29.3%)
Can stent be inserted using a catheterization	No	29 (2.9%)
	Yes	613 (62.2%)
	Don’t know	342 (34.7%)
Would you agree on a catheterization if needed?	No	45 (4.5%)
	Yes	771 (78.3%)
	Don’t know	168 (17.0%)

We attempted to assess the difference in perception of cardiac disease risk factors and symptoms between respondents who had at least one cardiac risk factor (diabetes, hypertension, dyslipidemia, or smoking) as compared to respondents who had no cardiac risk factors. It was counterintuitive to notice that more participants who had CAD risk factors had poor knowledge of heart attack symptoms, as shown in Table 5. Interestingly, when assessing common cardiac risk factors, there were no differences between the two groups except that respondents who had no cardiac risk factors were less likely to think that diabetes is a recognized risk factor for cardiac disease (p-value=0.012). When we evaluated the knowledge of respondents on the most common cardiac symptoms, there were no differences except in nausea and vomiting. Respondents who had at least one cardiac risk factor were more likely to consider nausea and vomiting as a symptom of cardiac disease (p-value= 0.002) (Table 5).

**Table 5:** Comparison of responders with at least one risk factor with those who have none *p-value at level of significance <0.05

Participants Who Have At Least 1 Cardiac Risk Factor		Yes, n= 357 (%)	No, n= 626 (%)	Total, n=984 (%)	P-value
Risk Factors					
	Diabetes is a Risk Factor	131 (36.7%)	185 (29.6%)	316 (32.1%)	<0.05*
	Hypertension is a Risk Factor	232 (65%)	374 (59.7%)	606 (61.6%)	0.1
	Dyslipidemia is a Risk Factor	263 (73.7%)	440 (70.3%)	703 (71.5%)	0.26
	Age is a Risk Factor	126 (35.3%)	244 (39%)	370 (37.6%)	0.25
	Overweight is a Risk Factor	229 (64.1%)	389 (62.1%)	618 (62.9%)	0.53
	Smoking is a Risk Factor	252 (70.6%)	413 (66.1%)	665 (67.7%)	0.15
	Sedentary Lifestyle is a Risk Factor	173 (48.5%)	282 (45%)	455 (46.3%)	0.3
	Unhealthy Diet is a Risk Factor	94 (26.4%)	175 (28%)	269 (27.4%)	0.6
	Genetics is a Risk Factor	82 (23%)	153 (24.4%)	235 (23.9%)	0.6
	Anger is a Risk Factor	156 (43.7%)	240 (38.3%)	396 (40.3%)	0.09
Symptoms					
	Chest Pain	267 (74.8%)	474 (75.7%)	741 (75.4%)	0.7
	Dizziness	119 (33.3%)	209 (33.4%)	328 (33.4%)	0.9
	Nausea and Vomiting	84 (23.5%)	99 (15.8%)	183 (18.6%)	<0.05*
	Pain in the Jaw or Arm	136 (38.1%)	225 (35.9%)	361 (36.7%)	0.5
	Palpitations	114 (31.9%)	225 (36%)	339 (34.5%)	0.2
	Pins and Needles in the Chest	202 (56.6%)	357 (57%)	559 (56.9%)	0.9
	Shortness of Breath	207 (58%)	382 (61%)	589 (59.9%)	0.4
	Sweating	154 (43.1%)	239 (38.2%)	393 (40%)	0.1

Because almost 40% of the respondents were health care workers, we sought to determine whether they have a better knowledge of coronary catheterization as compared to the general public. Table 6 demonstrates the responses to the questions with regard to coronary catheterizations. Non-healthcare workers had better knowledge on several points. Health care workers had a higher rate of choosing the “Do not Know” answer (Table 6). Additionally, more healthcare professionals will agree to have a cardiac catheterization if indicated (p-value= 0.005).

**Table 6:** Cath knowledge by being a health care worker or not *p-value at level of significance <0.05

Health Care Worker		Yes, n= 379 (%)	No, n= 605 (%)	Total, n=984 (%)	p-value
Heard of Cardiac Cath		365 (96.3%)	581 (96%)	946 (96%)	0.8
Knows the difference between diagnostic and therapeutic cath		89 (23.5%)	211 (34.9%)	300 (30.5%)	<0.05*
Who does the cath					
	Any Doctor	23 (6.1%)	35 (5.8%)	58 (5.9%)	0.5
	Cardiac Surgeon	181 (47.8%)	285 (47.1%)	466 (47.4%)	
	Interventional Cardiology	55 (14.5%)	108 (17.9%)	163 (16.6%)	
	Don’t know	120 (31.7%)	177 (29.3%)	297 (30.2 %)	
Use of contrast during a cardiac cath					
	Yes	98 (25.9%)	176 (29.1%)	274 (27.8%)	<0.05*
	Don’t know	252 (66.5%)	356 (58.8%)	608 (61.8%)	
Use of imaging during a cardiac cath					
	Yes	141 (37.2%)	254 (42%)	395 (40.1%)	0.054
	Don’t know	217 (57.3%)	302 (49.9%)	519 (52.7%)	
Cath can be done as a day case					
	Yes	127 (33.5%)	258 (42.6%)	385 (39,1%)	<0.05*
	Don’t know	114 (30.1%)	175 (28.9%)	289 (29.4%)	
Coronary Stent can be inserted during an angiogram					
	Yes	210 (55.4%)	403 (66.6 %)	613 (62.3%)	<0.05*
	Don’t know	114 (30.1%)	175 (28.9%)	289 (29.4%)	
Will you agree to have angiogram if needed					
	Yes	296 (78.1%)	475 (78.5%)	771 (78.4%)	<0.05*
	Don’t know	56 (14.8)	112 (18.5%)	168 (17.1%)	

**Figure 5:**
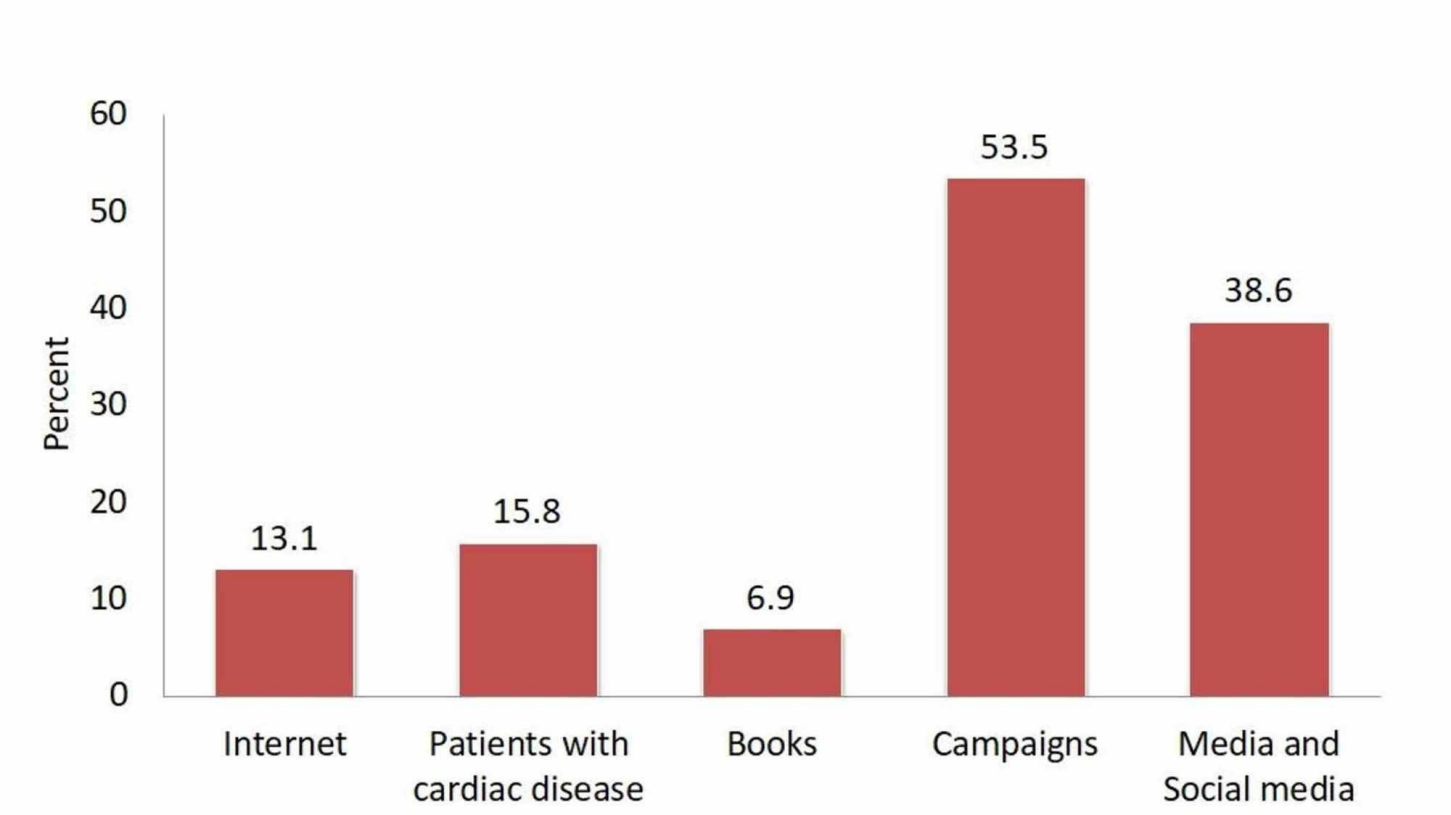
Source of coronary artery disease knowledge

Finally, participants were required to choose the source of information about CAD and cardiac catheterization. Fifty-three point five percent received their knowledge from health awareness campaigns while only 6.9% received their information from books, as shown in Figure 5.

## Discussion

The young population of Saudi Arabia is undergoing significant economic changes that have translated into the adoption of a Western dietary lifestyle that has led to a significant increase in the burden of cardiovascular diseases [17]. However, adequate baseline knowledge of CAD risk factors is lacking. Improved public awareness is associated with greater personal awareness and increased actions to lower CVD risk [18]. Knowledge related to heart disease has been associated with health promotion behaviors [19-20]. The study demonstrated fair knowledge of CAD risk factors and the role of coronary catheterization.

The majority of participants with or without CAD risk factors assumed that DM is the least likely to lead to the development of CAD. Participants without risk factors had significantly lower knowledge of the association between DM and CAD (p-value= <0.05). In contrast, many have recognized that dyslipidemia, hypertension, and smoking are risks for developing CAD. The study demonstrated poor knowledge of heart attack symptoms among patients with risk factors for CAD. While healthcare workers are more likely to accept undergoing coronary catheterization when required, they, had lower knowledge of aspects of cardiac catheterization as compared to non-healthcare professionals. Awareness campaigns seemed to be the most favorable source of information when learning about coronary heart disease and coronary catheterization among the population studied.

Almalki et al., in a recent study from Jeddah, reported a deficient level of awareness of CAD risk factors, DM, smoking, and lack of physical activity were identified by only 12%, 26%, and 39% of participants, respectively [15].

De Oliveira et al., in a small study, showed that patients were perplexed in differentiating between therapeutic and diagnostic coronary angiograms. They have reported heightened fear and anxiety among patients who had coronary catheterization; this was attributed to a lack of adequate knowledge of the procedure [21].

On the other hand, Tait et al., in a study that included 151 patients who underwent a pre-catheterization educational program, reported an improved understanding of the procedure, which resulted in better patient experience [22]. It is clear that educational programs are needed to spread knowledge and enhance the public awareness of the risk factors of CAD and its treatment in order to be effective in reducing the overwhelming burden of this devastating disease.

Limitations

The present study has many limitations: voluntary self-reporting of risk factors, lack of questioner validation, and high response from the younger age group with a high level of education, which might not be representative of the entire social spectrum. All these factors limit the generalizability of the survey.

## Conclusions

CAD disease is a significant health issue globally. Prevention can be achieved through the better control of the risk factors of atherosclerosis. Improved public knowledge will favorably contribute to the adoption of a better lifestyle that lessens the burden of CAD. Our study showed an explicit limited knowledge of CAD risk factors, symptoms, methods of diagnosis, and treatment among the Western population of Saudi Arabia. Additionally, it showed a lack of proper awareness of the role of coronary angiography and coronary interventions. Decision-makers in the Kingdom of Saudi Arabia are required to enhance public awareness through campaigns and screening programs.
