# Correction: The Perception of Coronary Artery Disease and Cardiac Catheterization in Saudi Arabia: “What the Public Know”

**DOI:** 10.7759/cureus.c27

**Published:** 2020-01-07

**Authors:** Saad Albugami, Faisal Al-Husayni, Lama Bakhsh, Faisal Alhameed, Ahmad Alsulami, Khalid Abumelha, Marwan Balubaid, Maha Al-Harbi, Hani N Mufti

**Affiliations:** 1 Cardiac Sciences / Interventional Cardiology, King Faisal Cardiac Center, King Saud Bin Abdulaziz University for Health Sciences / King Abdullah International Research Center, Jeddah, SAU; 2 Internal Medicine, National Guard Hospital, Jeddah, SAU; 3 Internal Medicine, King Saud Bin Abdulaziz University for Health Sciences, Jeddah, SAU; 4 Nursing, National Guard Hospital, Jeddah, SAU; 5 Cardiac Sciences / Cardiac Surgery, King Faisal Cardiac Center, King Saud Bin Abdulaziz University for Health Sciences / King Abdullah International Research Center, Jeddah, SAU; 6 Research Unit, King Abdullah International Medical Research Center, Jeddah, SAU; 7 College of Medicine, King Saud Bin Abdulaziz University for Health Sciences, Jeddah, SAU

Figure 5 was initially portrayed as a pie chart, but has been updated to a bar chart as the percentages exceed 100% and this is in line with the other charts used in the study.

